# Macular Amyloidosis and Epstein-Barr Virus

**DOI:** 10.1155/2016/6089102

**Published:** 2016-02-14

**Authors:** Yalda Nahidi, Naser Tayyebi Meibodi, Zahra Meshkat, Narges Nazeri

**Affiliations:** ^1^Skin Research Center, School of Medicine, Mashhad University of Medical Sciences, Mashhad 9176699199, Iran; ^2^Virology Research Center, School of Medicine, Mashhad University of Medical Sciences, Mashhad 9176699199, Iran; ^3^Pathology Department of Mashhad University of Medical Sciences, Mashhad 9176699199, Iran

## Abstract

*Background*. Amyloidosis is extracellular precipitation of eosinophilic hyaline material of self-origin with special staining features and fibrillar ultrastructure. Macular amyloidosis is limited to the skin, and several factors have been proposed for its pathogenesis. Detection of Epstein-Barr virus (EBV) DNA in this lesion suggests that this virus can play a role in pathogenesis of this disease.* Objective*. EBV DNA detection was done on 30 skin samples with a diagnosis of macular amyloidosis and 31 healthy skin samples in the margin of removed melanocytic nevi by using PCR.* Results*. In patients positive for beta-globin gene in PCR, BLLF1 gene of EBV virus was positive in 23 patients (8 patients in case and 15 patients in the control group). There was no significant difference in presence of EBV DNA between macular amyloidosis (3.8%) and control (23.8%) groups (*P* = 0.08).* Conclusion*. The findings of this study showed that EBV is not involved in pathogenesis of macular amyloidosis.

## 1. Introduction

**Figure 1:**
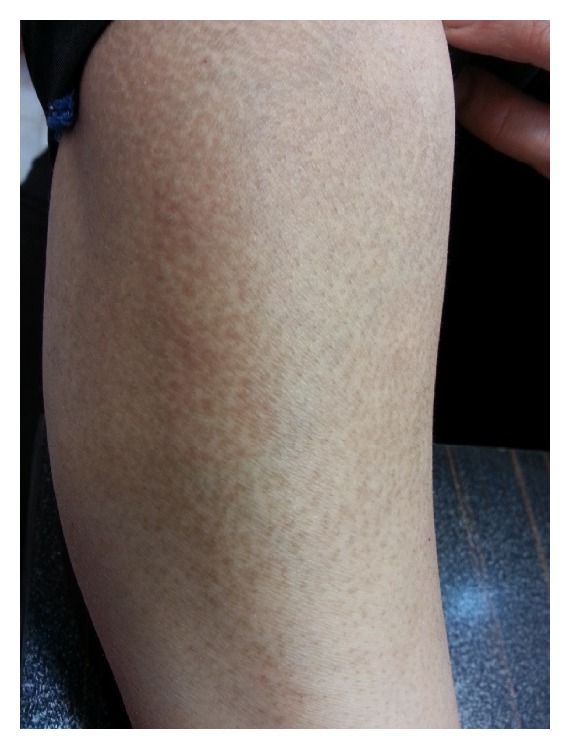
Hyperpigmented patch composed of small brown macules in a rippled or reticulated pattern on the arm.

Amyloidosis is the extracellular deposition of a group of fibrous proteins. There is a variety of approaches for classification of amyloidosis, but the simplest method is division into systemic and organ-specific (localized) types. Skin is an involved tissue in both types. Cutaneous localized amyloidosis is of two types: (i) keratinic, which may be primary or secondary, and (ii) nodular. The secondary type is secondary to other skin lesions such as skin tumors, inflammatory skin disorders, and phototherapy. Two types of keratotic amyloidosis have been identified: macular and lichen amyloidosis, and the latter is more common. In keratotic amyloidosis, keratin depositions originating from basal keratinocyte are mainly CK5 positive. Keratotic type is mostly observed in South-East Asia, South America, and China. It has familial (10%) and sporadic forms, and the latter is more common in women [1]. The most common sites of involvement are upper back (interscapular area) and extremities (shins and arms), although they have also been described on face, trunk, and thighs [2]. Macular amyloidosis lesions usually appear in the form of hyperpigmented patches with indefinite margins composed of grayish brown macules, often with a reticulated or rippled appearance [3] (Figure 1). Itching is a common symptom before the onset of amyloidosis [1].

**Figure 2:**
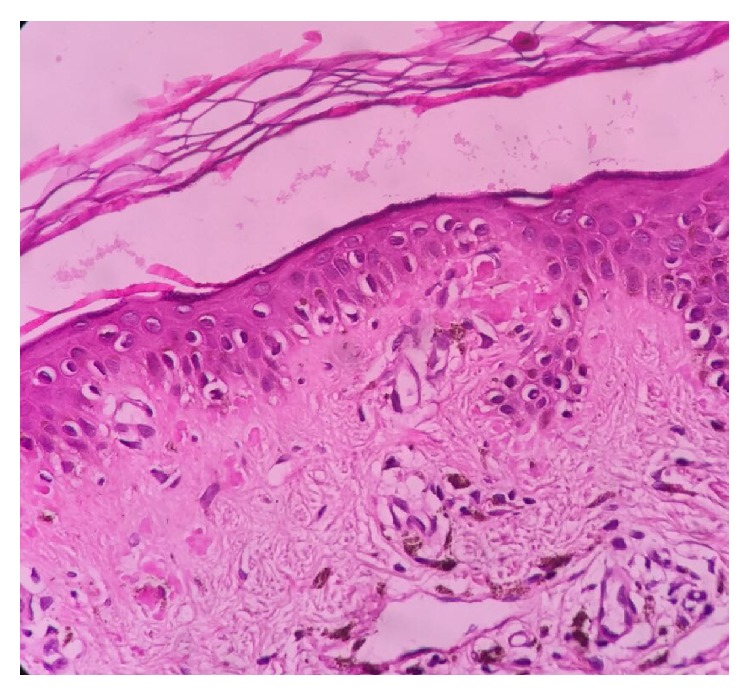
Small globular hyaline materials of amyloid in the papillary dermis.

The pathogenesis of this disorder is not fully elucidated, except for the fact that the deposited keratin is derived from keratinocytes [1]. Two pathogenic mechanisms have been proposed including apoptotic (fibrillar) theory and secretory theory [4]. Based on apoptotic theory, degeneration of damaged keratinocytes in the basal layer is followed by the conversion of these colloid bodies by dermal histiocytes and fibroblasts into amyloid in the papillary dermis [5] (Figure 2). According to the secretory theory, deposits of amyloid derived from degenerated basal keratinocytes spread into papillary dermis through the damaged lamina densa [6]. There are several etiologic factors implicated in the pathogenesis of macular amyloidosis: racial factors [7], genetic predisposition, environmental factors [8], sex (female) [9, 10], female hormones [11], exposure to sunlight [12], friction (long term abrasion) [12–14], atopy [15], autoimmunity (based on association with systemic lupus erythematosus, dermatomyositis, systemic sclerosis, sarcoidosis, and IgA nephropathy) [16, 17], and infection with EBV [18, 19]. EBV, the presence of which has been reported in epidermis of macular amyloidosis, is likely to act as a factor contributing to degeneration of keratinocytes [1].

The role of EBV in etiopathogenesis of primary cutaneous amyloidosis has been evaluated in only two prior studies including a single case report [18] and a case series of 27 patients from China [19]. Considering the shortage of studies on the association between EBV and macular amyloidosis, we set out to perform a study in this regard in Iran. Perhaps antiviral agents can be used in the future for treatment of this disease in case of association with EBV.

## 2. Materials and Methods

In this case-control study, 38 macular amyloidosis samples and 38 healthy skin samples around excised melanocytic nevi from age- and sex-matched patients without macular amyloidosis were enrolled in the clinical examination based on a nonrandom objective-oriented sampling. Inclusion criteria included paraffin blocks with sufficient tissue in archives of Pathology Department of Imam Reza Hospital in Mashhad diagnosed with macular amyloidosis based on pathology report and clinical presentation. Exclusion criteria included blocks with imperfect data in records and insufficient samples for PCR.

In the case group, amyloid deposition was confirmed by optical microscopy and Congo red staining. In the next step, six 5 *μ*m sections were prepared from each of the blocks in case and control groups in sterile conditions using sterile blade and were placed in sterile Eppendorf tubes.

### 2.1. Deparaffinization

Xylol/ethanol was used to deparaffinize the paraffinized tissues. One mL xylol was added to microtubes containing tissue sections and incubated in room temperature for half an hour with constant shaking. In the next step, microtubes were centrifuged in 13,000 rpm for 10 minutes, and their supernatant was discarded. These two steps were repeated once. Five hundred *μ*L of 100% ethanol was added to the precipitate and centrifuged for 10 m in 13,000 rpm after several inversions of microtube, and the supernatant was then removed. This step was repeated again. Finally, the resulting precipitate was placed in room temperature to completely evaporate ethanol but not the precipitate.

### 2.2. DNA Extraction

DNA extraction was done using BIO BASIC INC (Canada) kit with lot number 8401-140116. Lysis buffer-T was used for extraction. In the next step, 100 *μ*L extraction buffer and 10 *μ*L proteinase K were added to each microtube and were mixed. Tissue samples were added to the mixture and incubated at room temperature for 10 minutes. Then, the samples were incubated at 95°C for 3 minutes to inactivate proteinase K. One hundred *μ*L Universal Buffer NST was added to the tubes and inverted 10 times. The obtained mixture was used for PCR.

### 2.3. PCR

In this study, PCR was used to detect the presence of EBV genome in macular amyloidosis. After DNA extraction from paraffinized blocks, the quality of DNA extracted from paraffin-embedded tissues was determined using beta-globin gene primers. GH20 and PC04 beta-globin gene primers used in this study amplified a 260 bp fragment. The sequence of these primers was as follows:

 GH20: 5′ GAA GAG CCA AGG ACA GGT AC 3′.
 PC04: 5′ CAA CTT CAT CCA CGT TCA CC 3′.

The samples producing the 260 bp fragment using the desired primers were considered favorable for amplification of EBV virus BLLF1 gene.

Presence of EBV sequence in the extracted DNA samples was tested using Cinna Gen kit with lot number 935701 (Sina Clon, Iran). This kit has been designed to determine the quality of EBV DNA in infected samples using PCR. Optimized 1x PCR as a mixture of recombinant* Taq* DNA polymerase, PCR buffer, MgCl_2_, dNTPs, and primers was the reagent used for mixing. The highly specific and repetitive region of BLLF1 gene encoding gp 350/220 is amplified by primers. They can detect at least 30 copies of EBV. The presence of 239 bp or 256 bp fragments indicates a positive test result.

Data analysis was performed using SPSS 11.5 software. Graphs and statistical tables were used to describe data and Chi-square and independent *t*-tests were used to compare EBV in healthy and patients' samples. In all tests, the significance level of 0.05 was considered.

## 3. Results

Fifty percent of the patients were male (19/38) and 50% were female (19/38). Three patients were in the age group under 20 years (7.9%), 5 patients 20–30 years (13.2%), 10 patients 30–40 years (26.3%), 13 patients 40–50 years (34.2%), 4 patients 50–60 years (10.5%), and 3 patients over 60 years (7.9%). The minimum and maximum ages were 19 and 76 years, respectively. There were 27 cases of infection in the trunk (71.1%) and 11 cases in the extremities (28.9%). The study and control group were matched for age (*P* = 0.535) and sex (*P* = 0.646).

**Figure 3:**
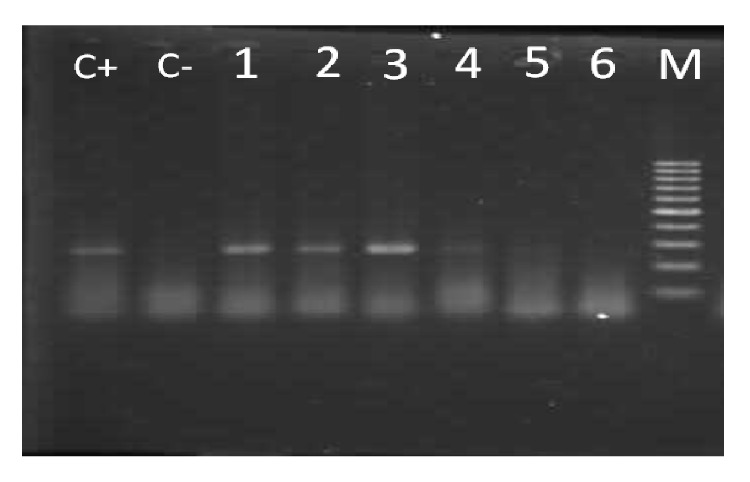
PCR results of beta-globin gene: in terms of amplification, samples 1, 2, and 3 are part of positive beta-globin gene (260 bp band) and samples 4, 5, and 6 are negative. C+ and C− indicate positive and negative controls, respectively, and M represents the DNA marker.

PCR was conducted for beta-globin gene in 76 samples (38 cases and 38 controls), which was positive in 61 samples (30 samples in case group and 31 in the control group) and was negative in 15 samples (8 samples in case and 7 in control group) (Figure 3).

**Figure 4:**
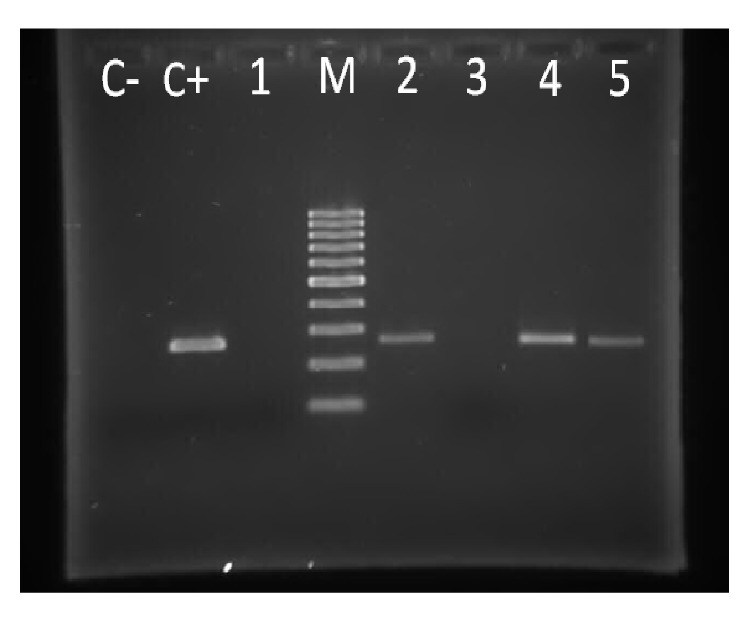
PCR results of BLLF1 gene of EBV: samples 2, 4, and 5 are positive (239 bp or 256 bp bands) and samples 1 and 3 are negative. C+ and C− indicate positive and negative controls, respectively, and M represents the DNA marker.

In 61 samples positive for beta-globin gene in PCR, BLLF1 gene from EBV was positive in 23 cases (8 cases in study group and 15 cases in control group) and was negative in 38 cases (22 cases in the study group and 16 cases in the control group) (Figure 4).

PCR of BLLF1 gene from EBV was 26.7% positive in study group and 48.4% positive in control group. Chi-square test results showed no correlation between EBV and macular amyloidosis (*P* = 0.08) (Table 1).

## 4. Discussion

Amyloidosis is known as extracellular deposition of eosinophilic hyaline material of self-origin with specific staining and ultrastructural characteristics. This disorder can occur in the background of systemic diseases or may be limited to the skin. Macular amyloidosis is limited to the skin [1]. EBV may stimulate the secretion of amyloid material by keratinocytes or may be a stimulus for degeneration of keratinocytes and conversion of degenerated keratinocyte filaments into amyloid [1, 18]. Recent studies have indicated the role of epithelial cells in continued reproduction of EBV. EBV cell surface receptors found in less differentiated squamous epithelium suggest the direct infection of epidermal keratinocytes. Infection may occur in germinative layers; however, virus replication is only feasible through maturation and differentiation of cells. The expression of cytokeratin in human keratinocytes is changed in vitro after infection with EBV, resulting in their conversion to fibroblasts [18, 19]. Fibroblasts can phagocytize keratin aggregates and convert them to amyloid [18].

Drago et al. showed this correlation in Italy in 1996. Their patient was a 30-year-old female with a ten-year history of itchy brown papules and macules on the chest and back with symptoms of chronic fatigue syndrome. They could show EBV genome in epidermal lesions using in situ hybridization technique. EBV genome was principally shown in basal epidermal cells as well as higher layer cells, especially in the cytoplasm. Serological tests for EBV were also positive in the patient. Antiviral therapy with acyclovir and interferon alpha improved skin lesions and general symptoms in the patient [18]. Another study was conducted by Chang et al. on skin tissue of 27 patients with a diagnosis of lichen and macular amyloidosis in Taiwan in 1997. In situ hybridization method indicated EBV DNA in lesions of 11 patients (40.7%), while the control group (including three patients with secondary cutaneous amyloidosis, two patients with primary systemic amyloidosis, and four patients with chronic simplex lichen) lacked EBV DNA [19].

According to this study, there was no correlation between EBV and macular amyloidosis (*P* = 0.08). EBV DNA was present in 8 patients with macular amyloidosis and 15 controls in our study. The difference in rate of detection of EBV DNA between macular amyloidosis patients and controls in this study with the mentioned studies can be due to the following reasons:

1. Lack of association between macular amyloidosis and EBV infection: there are a few studies presenting insufficient evidence for a definitive correlation between EBV and macular amyloidosis, so based on the results of our study we could make this conclusion.
2. Methodology (PCR versus in situ hybridization): we used a sensitive method for detection of EBV DNA with positive and negative controls. Based on the previous studies, PCR is as sensitive as in situ hybridization [20, 21]. However, as we did not use in situ hybridization, we could not localize the exact infected cell with EBV in our controls which might be the circulating B cells of the skin instead of keratinocytes. As we used positive and negative controls in our PCR kit for EBV, the positive cases in our control group could not be false positive.
3. Controls: the controls in our study were the healthy skin around melanocytic nevi, but the controls in Chang study were other cutaneous disorders.
4. Type of cutaneous amyloidosis: in our study, all the patients had macular amyloidosis, but in both the previous studies [18, 19] most of the patients were of lichen amyloidosis.

## 5. Conclusion

According to the results of this study, there was no correlation between EBV and macular amyloidosis. We recommend the use of fresh tissue or quickly frozen biopsy punch as well as simultaneous serological study of patients for anti-EBV antibody to achieve more accurate results for comparative study of EBV DNA in macular amyloidosis. In situ PCR can also be used to localize the EBV DNA positive cells in the samples. Other genes can be used to detect EBV in the tissue since some EBV samples are mutated for BLLF1 gene used in this study [22].

Also, we recommend performing a comparative study on detection of EBV in both involved and uninvolved skin of the patients with macular amyloidosis.

## Figures and Tables

**Table 1:** Percentage distribution of PCR results from BLLF1 gene of EBV in 30 patients with macular amyloidosis and 31 melanocytic nevus samples among samples positive for beta-globin gene.

EBV DNA PCR	Study groups				Chi-square test results
	Cases		Controls		
	Number	Percent	Number	Percent	
Positive	8	26.7	15	48.4	*P* = 0.08
Negative	22	91.7	16	76.2	

Total	30		31		61
